# Research on the impact of urban innovation on public health

**DOI:** 10.3389/fpubh.2024.1535932

**Published:** 2025-01-06

**Authors:** Liang Zhao, Chen Li, Yaosen Qian

**Affiliations:** ^1^School of Tourism, Hubei University, Wuhan, China; ^2^School of Management, Shanghai University of Engineering Science, Shanghai, China; ^3^School of Naval Architecture, Ocean and Energy Power Engineering, Wuhan University of Technology, Wuhan, China

**Keywords:** innovation, public health, number of doctors per 10,000 people, per capita fiscal expenditure on healthcare, Chinese cities

## Abstract

**Introduction:**

This article explores the impact of innovation on urban public health, with a particular focus on panel data from 15 sub-provincial cities in China. The study aims to provide scientific evidence for policymakers by analyzing how technological innovation affects urban public health levels.

**Methods:**

The study used a panel model for empirical analysis which based on panel data from 15 sub provincial cities across the country, using the number of doctors per 10,000 people and per capita financial medical and health expenditure as proxy variables for urban public health, and using the level of technological development as the core explanatory variable for regression analysis.

**Results:**

The research results show that: (1) for public health quantified by the number of doctors per 10,000 people in cities, innovation does not have a significant promoting effect on urban public health; (2) Compared to the number of doctors per 10,000 people in a region, the per capita financial expenditure on healthcare can better measure the level of urban public health; (3) Innovation has a significant impact on urban public health, measured by per capita fiscal expenditure on healthcare.

**Discussion:**

In order to transform technological innovation into a driving force for the development of urban public health, efforts must be made from multiple aspects. Currently, building a strong foundation for people’s health relies on the support of science and technology, and enhancing innovation as a primary driving force is crucial. China urgently needs to improve the stability and competitiveness of its pharmaceutical industry and supply chain, break through key core technologies, and take the initiative in the future development of the pharmaceutical industry.

## 1 Introduction

The healthcare industry has always been closely linked with the overall national strategy and plays an important supporting role. People’s health is an important symbol of China’s modernization, and safeguarding people’s health cannot be separated from technological support. On the new journey of comprehensively building a socialist modernized country and promoting the construction of a healthy China, it is necessary to further popularize healthy living, optimize health services, improve health guarantees, build a healthy environment, and develop the health industry. The Chinese government places the protection of people’s health in a strategic position of priority development, accelerates the construction of a healthy China, and explicitly requires innovation to be “oriented towards people’s lives and health.” History and reality have fully proven that the development of the healthcare industry must rely on innovation to lead and promote, and ensuring human health cannot be separated from scientific development and technological innovation.

Science and technology are the most powerful weapons for humans to fight against diseases (1–3). The victory of humanity over major disasters and epidemics cannot be achieved without scientific development and technological innovation. Currently, the demand for a healthy lifestyle among the people is constantly increasing, and the status of the healthcare industry in the national strategy is also constantly rising. People’s multi-level and diversified health needs will continue to grow rapidly, and the country will also put forward higher requirements for medical innovation (4–8). Compared to this, China’s medical innovation system has not yet formed an overall advantage, and there are still shortcomings in key aspects such as the ability and output of medical innovation, the system and institutions, investment and support. Specifically, there is an urgent need to strengthen basic research capabilities and original innovation capabilities, improve the mechanism for coordinating scientific and technological resources and the independent and controllable innovation system, and enhance the guidance of medical innovation investment and direction. There is still a significant gap between China’s medical technology and advanced countries in terms of overall innovation system and innovation capability, and there is still a long way to go to achieve the goal of providing strong support for building a world science and technology power.

There is a close and dynamic relationship between innovation and public health. At present, research progress in this field is reflected in multiple aspects. One is about innovating and improving the level of public health management. Through big data analysis and artificial intelligence technology, health data can be collected, stored, and analyzed more effectively, helping medical institutions understand the health status of a certain area, predict and monitor the spread of diseases (9–12). Big data technology can also be applied to evaluate the built environment related to public health, such as processing street view images through machine learning algorithms, identifying human scale information, and planning and designing healthy living environments. Artificial intelligence technology has made significant progress in medical diagnosis, treatment, and surgical planning, improving the accuracy and efficiency of diagnosis (13–18).

The second is to innovate and promote the optimization of public health services. With the continuous development of artificial intelligence technology, personalized medicine and health management have become possible (19–21). By analyzing patients’ personal data and health records, personalized treatment plans and health management plans can be developed. Gene editing technologies such as CRISPR-Cas9 have also provided individuals with customized treatment plans, bringing revolutionary progress to medical technology. Medical robots have been widely used in fields such as surgery, rehabilitation, and nursing, improving the accuracy and stability of surgeries, reducing rehabilitation time, and enhancing rehabilitation outcomes (22–24). Bioprinting technology is an advanced technique that utilizes biomaterials and cells for directional printing in three-dimensional space to form tissues or organs. This technology has broad application prospects in the fields of tissue engineering and organ transplantation, providing the possibility for organ regeneration and transplantation.

The third is the challenges and response strategies faced by innovation. With the widespread application of big data and artificial intelligence technology, data privacy and security issues are becoming increasingly prominent (25–28). The response strategies include strengthening data encryption and access control, developing strict data privacy protection policies, and increasing public awareness and importance of data privacy. With the continuous advancement of technology, relevant laws, regulations, and ethical norms also need to be constantly improved. The response strategy includes strengthening the formulation and implementation of laws and regulations, establishing a reasonable ethical norm system, and enhancing international cooperation and sharing of experience. In addition, in the face of global public health challenges, international cooperation and sharing will become an important trend for future development. By strengthening international cooperation and sharing data resources, technological achievements, etc., we can jointly address global public health issues and improve the level of global public health (29–31).

In summary, innovation has made significant research progress in improving the level of public health management, promoting the optimization of public health services, and addressing challenges and response strategies. However, from the existing literature, research on the impact of innovation on public health is mostly interpreted from frameworks and concepts, with more qualitative research and less quantitative research. There is a lack of research literature on the impact mechanism of innovation on urban public health. Current research on the impact of innovation on public health is mostly interpreted at the framework and conceptual level, with more qualitative research and relatively less quantitative research. This means a lack of specific data analysis and empirical support to verify the actual effectiveness of innovation. In existing research literature, there is a lack of in-depth exploration of the specific mechanisms by which innovation affects urban public health. This limits our understanding of how innovation specifically affects the public health system, making it difficult to develop more effective policies and strategies. Although the application of big data analysis and artificial intelligence technology in public health management has been mentioned, existing research may still have shortcomings in providing practical data support. Lack of specific data analysis and case studies to demonstrate the practical application effects of innovative technologies.

In view of this, this article chooses innovation elements as the core explanatory variables, incorporates innovation and urban public health into a holistic research framework, focuses on measuring the level of public health development in 15 sub provincial cities in China, and explores the impact of innovation on urban public health. The article combines innovation, an important engine for regional economic development, with urban public health, a social welfare field, to explore the impact of innovation on the level of urban public health. This cross disciplinary perspective may not have been common in previous research, providing new ideas for understanding the social benefits of innovation. The article used panel data from 15 sub provincial cities across the country and systematically studied the relationship between innovation and urban public health through statistical methods such as regression analysis. This empirical research based on big data and statistical methods enhances the reliability and persuasiveness of the research results. When measuring the level of urban public health, the article not only considers the traditional indicator of the number of doctors, but also introduces the more comprehensive indicator of per capita fiscal medical and health expenditure. The innovation in variable selection enables research to more accurately capture the comprehensive impact of innovation on urban public health levels.

## 2 Variable selection and data explanation

### 2.1 Research on urban overview and data sources

This study selected 15 sub provincial cities across the country as research subjects. A sub provincial city is a provincial-level city under the administrative structure of sub provincial level in China, officially implemented in 1994. Its predecessor was a planned city. There are currently 15 sub provincial cities in China, namely Shenyang, Dalian, Changchun, Harbin, Nanjing, Hangzhou, Qingdao, Jinan, Ningbo, Xiamen, Wuhan, Chengdu, Xi’an, Guangzhou, and Shenzhen. Except for Dalian, Qingdao, Ningbo, Xiamen, and Shenzhen, all of these cities are provincial capitals. The reason for choosing sub provincial cities is that these cities have relatively good economic foundations, outstanding development, and special economic advantages that are different from mega cities. They also have coverage in the eastern, central, western, and northern and southern regions of the country, and therefore have certain representative significance. The relevant indicator data of each city mainly comes from the “China Urban Statistical Yearbook” (2008–2023) and the statistical yearbooks of each city.

### 2.2 Selection and explanation of variables

1. Urban Innovation: Urban innovation refers to the integration of internal and external resources, the use of new knowledge, technologies, and methods to create new economic growth points, social service models, or cultural forms in a constantly changing environment, in order to promote the sustainable development and progress of urban economy and society. This article takes innovation as the explanatory variable, and for the measurement indicators of innovation, Sun Yu et al. (2008) selected 18 indicators including per capita GDP, patent applications per 10,000 people, patent authorizations per 10,000 people, and the proportion of scientific expenditure to total fiscal expenditure as indicators of urban innovation capability (32); Yu Liping (33) used provincial R&D investment data, including full-time equivalent R&D personnel, technology market transaction volume, and number of invention patents as innovation indicators (33); Fan Jie and Liu Hanchu (34) selected 11 representative indicators based on the connotation of regional innovation capability, including per capita financial science and technology funding and the number of patent applications accepted per 10,000 people (34); Zhou Ke et al. (35) used the number of domestic patent applications granted in each province over the years to measure innovation (35). From the construction of indicators by scholars, it can be seen that innovation variables have mostly chosen indicators such as patent application (authorization) volume and fiscal technology expenditure. Drawing on the indicators constructed by scholars and based on the needs of this study and the availability of data, this article uses the proportion of technology expenditure to urban fiscal expenditure and the number of patent authorizations per 10,000 people in the city to measure innovation indicators. Meanwhile, as James and Ron (36) pointed out, economic diversity is believed to affect regional public health and adaptability through various pathways (36). Therefore, factors such as employment form diversity, industrial diversification, and imports and exports associated with this can all be used as control variables. This article uses the proportion of urban private and individual employees to the total employed population to represent employment diversity, the proportion of the tertiary industry to urban GDP to represent industrial diversity, and the import and export variables are expressed as the ratio of the city’s annual import and export volume to the city’s GDP. The import and export volume is converted into RMB based on the exchange rate of the current year.
2. The public health level of the city: Urban public health (Phenath). The article is consistent with most literature, focusing on the health status of urban residents in terms of physical function, while taking into account the necessary basic support conditions and practical health performance improvement when measuring public health to construct a composite index of urban public health. The necessary foundation of public health is the logical starting point and prerequisite for improving urban public health, represented by the number of doctors per 10,000 people and per capita financial medical and health expenditures.

## 3 Empirical analysis

### 3.1 Basic model

The basic research model of this article is established as follows:

(1) Yit=αit+βitXit+θitVit+εit

In formula (1), *Y_it_* is the dependent variable representing public health, and *X_it_* is the core explanatory variable representing innovation indicators for each city in different years. These indicators are represented by the ratio of science and technology expenditure to fiscal expenditure (*texp*) and the number of patent authorizations per 10,000 people in each city. Vit represents control variables, including employment diversity (*empl*), industry diversity (*serind*), and the proportion of total import and export value to GDP (*imex*). Due to the fact that the dependent variable public health has two variables—the number of doctors per 10,000 people (*nodp*) and the per capita fiscal healthcare expenditure (*unem*), we constructed Model 1 and Model 2 for analysis, respectively (Table 1).

**Table 1:** Hausman test results.

	Number of doctors per 10,000 people (*nodp*)	Per capita fiscal expenditure on healthcare (*pceh*)
*Chi-Sq. Statisic*	70.6271	1.0089
*Chi-Sq. d.f.*	5	5
*Prob.*	0.0000	0.9618

### 3.2 Construction and analysis of model 1

To confirm whether the panel model is suitable for a random effects model or a fixed effects model, the Hausman test method was used. The null hypothesis is that a random effects model should be established. According to the Hausman test results shown in Table 2, for the urban *grp* index, the value of the Hausman statistic is 70.6271, the degree of freedom is 5, and the corresponding *p*-value is 0.0000, which is less than 0.05. This rejects the null hypothesis of the random effects model, so a fixed effects model should be chosen; For the unemployment rate indicator, the Hausman statistic has a value of 1.0089, a degree of freedom of 5, and a corresponding *p*-value of 0.9618, which is greater than 0.05. Therefore, the null hypothesis of the random effects model should be accepted, and the unemployment rate model should choose the random effects model.

**Table 2:** Model 1 regression results.

Explanatory variable	Proportion of technology investment in fiscal expenditure(*texp*)	Thousands of people have patents authorization quantity(*pate*)	Diversity in employment(*empl*)	Industrial diversity(*serind*)	Proportion of imports and exports(*imex*)
Shenyang	0.1989(0.0372)	−0.0220***(5.4004)	0.1334(0.5454)	−0.5449*(−1.8795)	0.0765(0.1630)
Dalian	5.7121(1.4886)	−0.0071(1.6617)	−0.5828(−1.6216)	−0.1634(−0.3392)	0.1330(0.9444)
Changchun	3.1766(0.6920)	−0.0198**(2.3232)	0.4521(1.1420)	0.7192(0.7472)	0.6555*(1.9421)
Haerbin	0.4474(0.0838)	−0.0068(1.1135)	0.1543(0.9687)	−0.4862(−0.3581)	−0.6866(−0.8948)
Nanjing	−2.0985 (0.6236)	0.0027(0.8580)	−0.2246(−0.8593)	−1.0878(−0.8750)	−0.0402(−0.2245)
Hangzhou	−4.3562(1.2761)	0.0009(0.8312)	0.3093(1.1204)	0.0415(0.1192)	0.0174(0.0868)
Qingdao	−1.8008(0.6966)	0.0012(0.3765)	−0.0793(−0.2288)	−0.6772(−0.4795)	0.0361(0.6619)
Jinan	−1.3587(0.3215)	−0.0010(0.4428)	−0.0437(−0.4638)	−0.5402(−0.7591)	0.1594(0.8771)
Ningbo	2.9456(1.3441)	−0.0001(0.3433)	−0.7259***(−2.7072)	0.6322(0.8174)	−0.0312**(−2.0627)
Xiamen	−11.0347**(2.2434)	0.0006(0.8303)	0.0521(0.1780)	−0.7464(−1.1729)	0.1140(0.7893)
Wuhan	−1.4258(1.2993)	0.0020(0.4531)	−0.3353(−0.8958)	−0.2439(−0.4834)	0.2082(0.7856)
Chengdu	−2.5262(1.1523)	−0.0033*(1.8285)	−0.0043(−0.0562)	1.0204(1.2441)	0.1570(1.0546)
Xian	0.9620(0.4029)	−0.0013(1.2444)	0.0262(0.1032)	−0.2376(−0.4346)	−0.1940(−0.6159)
Guangzhou	0.5018(0.6440)	0.0005(0.2299)	−0.2126(−1.1526)	−0.2120(−0.1776)	0.1021(0.7549)
Shenzhen	0.4439(1.3339)	0.0000(0.2430)	−0.0874(−0.4932)	−0.1560(−0.2074)	0.0346(1.0718)

The t-test is indicated in parentheses, with *, **, and ** representing significance levels of 10, 5, and 1%, respectively.

According to whether the intercept and coefficient are variable, fixed effects models can be divided into constant parameter models with constant intercept and coefficient, variable intercept models with constant coefficient and variable intercept, and variable coefficient models with variable intercept and coefficient. To determine the specific form that an individual’s panel fixed effects model should take, we construct two hypotheses:

*H*1: The coefficients of the model are all equal, that is, the proportion of technology expenditure to fiscal expenditure, the number of patent authorizations per 10,000 people, industrial diversity, employment diversity, and the proportion of total import and export value to GDP have the same impact on urban public health.

*H*2: The intercept of the model is equal, and the coefficients are also equal, assuming that the public health of each city is the same, and each indicator including the proportion of technology expenditure to fiscal expenditure, the number of patent authorizations per 10,000 people, industrial diversity, employment diversity, and the proportion of total import and export value to GDP have the same impact on urban public health.

Next, construct two F-statistics to test the above two hypotheses:


$$\begin{array}{l} F1=\frac{\left(S2-S1\right)/\left[\left(N-1\right)k\right]}{S1/\left(NT-N\left(k+1\right)\right)}\sim F[\left(N-1\right)k,N(T-k-1]\\ F2=\frac{\left(S3-S1\right)/\left[\left(N-1\right)\left(k+1\right)\right]}{S1/\left(NT-N\left(k+1\right)\right)}\sim F\left[\left(N-1\right)\left(k+1\right),N\left(T-k-1\right)\right] \end{array}$$

Through multiple regressions of *grp*, we can obtain *S_1_* (sum of squared residuals of variable coefficient model), *S_2_* (sum of squared residuals of variable intercept model), and *S_3_* (sum of squared residuals of constant parameter model) as follows:

*S_1_* = 0.0146, *S_2_* = 0.0871, *S_3_* = 0.1447.

So calculate *F_1_* and *F_2_* as follows:

*F_1_* = 4.2458, *F_2_* = 6.3488.

Firstly, test *H_2_*. The *F*-distribution value F_0.05_(84, 60) = 1.4970 compared to *F_2_* is smaller than *F_2_*. Then, test *H_1_*. The F-distribution value F_0.05_(70, 60) = 1.5160 compared to *F_1_* is smaller than *F_1_*. Therefore, for the public health indicator *nodp*, we should construct a fixed effects variable coefficient model, that is, formula (2) is:


(2) $$\begin{array}{l} \mathrm{nodp}=\alpha_{i}+\beta_{1i}\cdot \mathrm{texpit}+\beta_{2i}\cdot \mathrm{pateit}+\beta_{3i}\cdot \mathrm{emplit}\\ \;+\beta 4i\cdot \mathrm{serin}d_{\mathrm{it}}+\beta 5i\cdot imex_{\mathrm{it}}+u_{\mathrm{it}} \end{array}$$

The regression results are as follows:

From Table 3, it can be seen that from the perspective of the impact of innovation on the public health indicator *nodp*, the impact of innovation on urban public health is not significant. However, in a few cities with significant impact, such as Xiamen, there is a negative relationship between technology investment (texp) and urban public health indicators, which seems counterintuitive. However, from another perspective, it is not difficult to understand that the process from technology investment to output itself has a time lag effect, and technology investment cannot immediately improve public medical resources. In addition, from the perspective of the proportion of technology investment to fiscal investment in each city, this value is very small. For example, in Harbin, the proportion of technology investment to fiscal expenditure in 2016 was only 0.87%. Even in Hangzhou, where technology investment is relatively high, technology investment only accounted for 5.33% of fiscal expenditure in 2016. Meanwhile, from the innovation indicator of the number of patents granted to 20,000 people, we can also see that in most cities, the number of patents granted is not significantly related to urban public health. Only Shenyang, Changchun, and Chengdu have a relatively significant impact on urban public health, but this impact is also relatively small and negative. For example, in Shenyang, for every one unit change in patent authorization, the public health sector will experience a reverse change of 0.02 units; For every unit change in patent authorization in Changchun, public health will experience a reverse change of 0.02 units; In Chengdu, for every 1 unit change in patent authorization, the public health sector experiences a reverse change of 0.003 units. Of course, these changes are relatively minor, and it can be said that the number of patent authorizations per 10,000 people has no significant impact on urban public health. The regional distribution of medical resources in China is very uneven, with the highest quality doctor resources concentrated in big cities such as Beijing, Shanghai, and Guangzhou, or in eastern coastal provinces. The density of doctors is high, and the corresponding supporting facilities are also good. However, in other regions, especially in the Midwest and rural areas, there is a relative scarcity of medical resources. This regional difference may lead to insignificant effects of innovation in increasing the number of doctors. There are significant differences in the allocation of medical resources between urban and rural areas, with urban areas having relatively abundant medical resources while rural areas have relatively scarce medical resources. This difference may lead to limited effectiveness of innovation in increasing the number of doctors in rural areas.

**Table 3:** Regression results of Model 2.

Variable	Coefficient	Standard deviation	T statistic	*p* value
C	0.0521	0.0098	5.2872	0.0000
TEXP?	−0.2349	0.0612	−3.8357	0.0002
PATE?	−0.0001	0.0000	−2.1437	0.0337
EMPL?	−0.0482	0.0078	−6.1484	0.0000
SERIND?	0.0117	0.0188	0.6228	0.5344
IMEX?	−0.0006	0.0017	−0.3410	0.7336

Of course, it may also be due to the large number of patent authorizations in Chinese cities, but the quality still needs to be improved, especially the invention patents that serve as the gold standard still need to be strengthened. In addition, it also indicates that the rate of patent conversion into production capacity in China is relatively low. Patents still mostly remain at the level of research and development. From other control variables, the impact of other factors on urban public health is also not the same. In Ningbo, both employment diversity and the proportion of imports and exports have a significant impact on the city’s public health. Of course, the impact is negative, meaning that the more employment diversity increases, the lower the city’s public health value; The larger the proportion of exports, the lower the public health value of the city, which may be related to the special geographical conditions of Ningbo. Ningbo is the world’s third largest port city, and its own private commerce is also exceptionally prosperous. After the economy reaches a certain level, it may be weakened or even alienated by the diversity of employment and import and export factors.

### 3.3 Construction and analysis of model 2

Similarly, for Model 2, we can calculate that:

*S_1_* = 0.0016, *S_2_* = 0.0044, *S_3_* = 0.0136


$$F1=\frac{\left(S2-S1\right)/\left[\left(N-1\right)k\right]}{S1/\left(NT-N\left(k+1\right)\right)}\sim F[\left(N-1\right)k,N(T-k-1]$$

$$F2=\frac{\left(S3-S1\right)/\left[\left(N-1\right)\left(k+1\right)\right]}{S1/\left(NT-N\left(k+1\right)\right)}\sim F\left[\left(N-1\right)\left(k+1\right),N\left(T-k-1\right)\right]$$

So we can calculate *F_1_* = 1.4179, *F_2_* = 5.1231.

First, check *H_2_*. Since F_0.05_(84, 60) = 1.4970 is less than *F_2_*, reject *H_2_*. However, F_0.05_(70, 60) = 1.5160 is greater than *F_1_*. Therefore, a variable intercept model for random effects should be constructed, and the expression for formula (3) can be obtained as follows:


(3) $$\begin{array}{l} \mathrm{unemit}=\alpha +\alpha_{i}+\beta 1\cdot \mathrm{texpit}+\beta 2\cdot \mathrm{pateit}+\beta 3\cdot \mathrm{emplit}\\ \;+\beta 4\cdot \mathrm{serin}d_{\mathrm{it}}+\beta 5\cdot imex_{\mathrm{it}}+u_{\mathrm{it}} \end{array}$$

The estimated values obtained are:

From the perspective of the impact of innovation on per capita fiscal healthcare expenditure (*unem*), innovation has a significant impact on urban public health. This indicates that providing strong technological support for “people’s life and health” requires increased investment, which is comprehensive and includes not only economic investment but also investment in talent teams. It should be noted that the rapid development of medical technology in the world has increasingly significant impacts on people’s health, economic development, and national security. These objective circumstances require our investment to be dynamic and continuous. In the future, a normalized national medical and health science fund can be established to focus on supporting clinical medicine, public health, translational medicine research, and basic scientific research work, further increasing the proportion of scientific and technological investment in the medical field in the overall national scientific and technological investment. The use of variable intercept in Model 2 means that although innovation has different impacts on various cities, the amount of change in innovation has the same impact on different regions. Every city can see the impact of innovation on its public health (Table 4).

**Table 4:** Estimated value of*α_i_*.

Number	City *i*	Estimated value of α	number	City *i*	Estimated value of α
1	Shenyang	0.0079	9	Ningbo	0.0023
2	Dalian	0.0096	10	Xiamen	−0.0100
3	Changchun	0.0073	11	Wuhan	0.0021
4	Haerbin	0.0012	12	Chengdu	−0.0100
5	Nanjing	−0.0012	13	Xian	0.0024
6	Hangzhou	−0.0121	14	Guangzhou	0.0147
7	Qingdao	−0.0028	15	Shenzhen	−0.0012
8	Jinan	−0.0102			

## 4 Conclusion and recommendations

This article uses a panel model to empirically test the impact of innovation on urban public health in 15 sub provincial cities in China. The conclusion is as follows: firstly, in terms of public health quantified by the number of doctors per 10,000 people in cities (*nodp*), innovative indicators have not shown a significant promoting effect, and some cities may even have a negative effect; Secondly, innovation has a significant impact on public health, which is quantified by per capita fiscal expenditure on healthcare (*unem*). The city’s technology investment (*texp*) and patent authorization (*pate*) both have a positive effect on the city’s public health. Of course, efforts need to be made from multiple aspects to better transform technology into momentum and maintain the development of urban public health. Now, more than ever before, building a strong foundation for people’s health requires the support of science and technology, and the enhancement of innovation as the primary driving force. Make due contributions to building a national medical innovation system, leading and promoting the development of the health industry, and constructing a human health community, continuously advancing toward the breadth and depth of science and technology.

In recent years, countries have increasingly attached importance to the strategic position of the pharmaceutical industry, and the global industrial chain and supply chain have been rapidly reshaped. China also urgently needs to improve the stability and competitiveness of the pharmaceutical industry and supply chain. Promote key core technology breakthroughs and seize the initiative in the future development of the pharmaceutical industry. Concentrate efforts to carry out key core technology research and accelerate the resolution of bottleneck problems in a number of fields such as drugs, medical devices, medical equipment, vaccines, etc. (6–14). Only by achieving independent and controllable key core technologies can we firmly grasp the initiative of innovation and development in our own hands.

Continuously increasing investment in technology. Technology investment is an important guarantee for promoting innovation and an important indicator for measuring innovation level and capability. Cultivate and strengthen innovation platforms. Innovation platforms are important carriers for gathering various innovative resources and elements, which help promote innovation to expand into deeper fields and move toward higher levels. Accelerate the upgrading of industrial structure, enhance the stability and competitiveness of the pharmaceutical industry supply chain. Accelerate the implementation of a batch of major research and development projects. We will continue to promote the development of innovative products through breakthroughs in innovative technologies, focusing on the needs of industrialization and bottleneck technologies. We will also develop innovative drugs, new vaccines, new antibody drugs, cell therapy, and gene therapy products. At the same time, we will accelerate the localization and substitution research of raw materials and key instruments and equipment, and strive to solve the bottleneck problem of key core technologies and raw materials. Actively creating a source of original technology. The development and integration of cutting-edge technologies and the improvement of people’s health awareness have promoted the rapid development of the global pharmaceutical industry, and also provided a broad space for China’s pharmaceutical industry to seize the opportunities of a new round of technological revolution and industrial transformation.

To improve the layout of scientific research, it is necessary to integrate the best medical research resources in the country, adopt a “center network” construction method in accordance with national strategic needs, optimize combination and system integration, and achieve full coverage of important medical disciplines such as basic medicine, clinical medicine, preventive medicine, public health, nursing, pharmacy, biomedical engineering, health management and policy. Combine the construction of the national medical innovation system with the establishment of national laboratories in the medical field, and continue to tackle major scientific and technological issues around serving national goals. At the same time, we will persist in conducting long-term and high difficulty research, cultivating innovative strategic forces that are at the forefront of global medical technology and can undertake national missions.

Based on the data of per capita fiscal medical expenditure, adjust the structure of fiscal expenditure and increase investment in the medical and health field to ensure that urban residents can obtain sufficient medical security. In response to the problem of uneven distribution of medical resources, the government should take measures to promote the balanced distribution of medical resources, such as increasing investment in medical and health care in rural or underdeveloped areas, and narrowing the medical gap between urban and rural areas and regions. The government can detect and solve problems in the medical and health field in a timely manner by monitoring and analyzing per capita financial medical expenditures, ensuring the effective utilization of medical resources and continuous improvement of medical services.

## Data Availability

Publicly available datasets were analyzed in this study. This data can be found at: https://www.stats.gov.cn.
